# Fast clearance of lipid droplets through MAP1S-activated autophagy suppresses clear cell renal cell carcinomas and promotes patient survival

**DOI:** 10.18632/oncotarget.6669

**Published:** 2015-12-18

**Authors:** Guibin Xu, Yaodong Jiang, Yuansong Xiao, Xian-De Liu, Fei Yue, Wenjiao Li, Xun Li, Yongzhong He, Xianhan Jiang, Hai Huang, Qi Chen, Eric Jonasch, Leyuan Liu

**Affiliations:** ^1^ Department of Urology, The Fifth Affiliated Hospital of Guangzhou Medical University, Guangzhou, Guangdong Province, 510700, China; ^2^ Center for The Innovation and Translation of Minimally Invasive Techniques, Guangzhou Medical University, Guangzhou, Guangdong Province, 510700, China; ^3^ Institute of Biosciences and Technology, Texas A&M Health Science Center, Houston, Texas, 77030, USA; ^4^ Department of Urology, Nanfang Hospital, Southern Medical University, Guangzhou, Guangdong Province, 510515, China; ^5^ Department of Urology, Guangzhou General Hospital of Guangzhou Military Command, Guangzhou, Guangdong Province, 510010, China; ^6^ Department of Genitourinary Medical Oncology, The University of Texas MD Anderson Cancer Center, Houston, Texas, 77030, USA; ^7^ Department of Molecular and Cellular Medicine, College of Medicine, Texas A&M Health Science Center, College Station, Texas, 77843, USA

**Keywords:** ADFP, ccRCC, lipophagy, MAP1S, prognosis

## Abstract

Clear cell renal cell carcinoma (ccRCC) is composed of cells whose cytoplasm filled with lipid droplets, subcellular organelles coated with adipocyte differentiation-related protein (ADFP) for the storage of triacylglycerol converted from excess free fatty acids. Mammalian cells primarily use the autophagy-lysosome system to degrade misfolded/aggregated proteins and dysfunctional organelles such as lipid droplets. MAP1S (originally named C19ORF5) is an autophagy activator and promotes the biogenesis and degradation of autophagosomes. Previously, we reported that MAP1S suppresses hepatocellular carcinogenesis in a mouse model and promoted the survival of patients with prostate adenocarcinomas by increasing the degradation of aggregated proteins and dysfunctional mitochondria. Here we show that a suppression of MAP1S in renal cells causes an impairment of autophagic clearance of lipid droplets. In contrast, an overexpression of MAP1S causes an activation of autophagy flux and a reduction of lipid droplets so less DNA double strand breakage is induced. The levels of MAP1S in normal renal cells are dramatically higher than those in the ccRCC tissues and cell lines derived from renal cell carcinomas. High levels of MAP1S are associated with a reduced malignancy and metastasis of ccRCC and predict a better survival of ccRCC patients. Therefore, autophagy defects in the degradation of lipid droplets triggered by the MAP1S deficiency may enhance the initiation and development of ccRCC and reduce the survival of ccRCC patients.

## INTRODUCTION

Mammalian cells primarily use the autophagy-lysosome system to degrade dysfunctional organelles, misfolded/aggregated proteins and other macromolecules [1, 2]. Defects in autophagy lead to an enhancement of oxidative stress [3, 4] or lysosomal rupture [5]. Reactive oxygen species cause telomere attrition and DNA double strand breakage [6, 7] and simultaneously subvert mitotic checkpoints [8, 9]. The resulting genomic instability is amplified through a cascade of autocatalytic karyotypic evolution through continuous cycles of chromosomal breakage-fusion-bridge and eventually leads to tumorigenesis [4, 10]. Furthermore, oxidative stress and lysosomal rupture in turn activates NLRP3 inflammasomes that result in a direct activation of caspase-1 [11]. The activation of caspase-1 eventually induces an inflammatory form of cell death referred to as pyroptosis [12]. The release of immunogenic danger signals or danger-associated molecular patterns (DAMPs) from pyroptotic cells can fuel pro-inflammatory cascades that promote the mortality of host structural, hematopoietic and immune-competent cells [13, 14]. Therefore, autophagy may be directly related to cancer initiation, development and metastasis as well as affecting survival of cancer patients.

MAP1S, previously named as C19ORF5, is a member of the microtubule-associated protein family 1. Similar to its homologues MAP1A and MAP1B, MAP1S interacts with both LC3-I and LC3-II isoforms [15–19]. We identified MAP1S as a positive regulator of autophagy and its depletion led to autophagic defects under nutritive stress and an accumulation of dysfunctional mitochondria [19]. Although no dramatic phenotype was observed, the general MAP1S knockout mice exhibited increased intensities of sinusoidal dilatation and increased levels of oxidative stress in liver, and reduced lifespans [20]. Based on the somatic mutation data of 17301 genes from 316 ovarian cancer patients from *The Cancer Genome Atlas*, MAP1S emerged as one the most significant patient survival-related genes [21]. When hepatocytes were exposed to chemical carcinogen, and when tumor cells in hepatocellular carcinomas were under metabolic stress induced by genomic instability, MAP1S was dramatically elevated and activated autophagy to degrade P62-associated protein aggregates and dysfunctional mitochondria, eliminate g-H2AX-labeled DNA double-strand breaks and suppress genomic instability. MAP1S-deficient mice developed more malignant hepatocellular carcinomas [22]. MAP1S levels were increased in response to the initiation and development of human prostatic adenocarcinomas (PCA), but subsequent loss of MAP1S expression in PCA patients led to shortening of their overall survival [23]. Therefore, an association between MAP1S-acitivated autophagic flux, malignancy and patient survival has been documented.

Clear cell renal cell carcinoma (ccRCC), the most common form of adult renal cell carcinomas (RCC), is composed of cells with clear or eosinophilic cytoplasm filled with lipids and glycogen, and arises from proximal tubular epithelial cells or other renal cell types. The cytoplasm contents are dissolved during routine histological processing and cells reveal a clear cytoplasm surrounding by a distinct cell membrane [24]. It is possible that defects in autophagy, especially defects in lipophagy which is the autophagic turnover of lipid droplets [25], may be responsible for the accumulation of lipid droplets in ccRCC. In our study, we found that normal renal cells in mouse and human renal tissues contained high levels of MAP1S, while ccRCC tissues and cell lines derived from RCC contained dramatically reduced levels of MAP1S. The suppression of MAP1S in renal cells caused an impairment of autophagic flux, an accumulation of lipid droplets and an increased genomic instability while its overexpression caused an activation of autophagic flux, a reduction of lipid droplets and a decreased genomic instability. High levels of MAP1S were associated with a reduced aggressivity of ccRCC and an improved survival of ccRCC patients. Therefore, defects in autophagic clearance of lipid droplets triggered by the MAP1S deficiency may stimulate the initiation, development and metastasis of ccRCC, and reduce the survival of ccRCC patients.

## RESULTS

### Distribution of MAP1S in renal tissues

**Figure 1:**
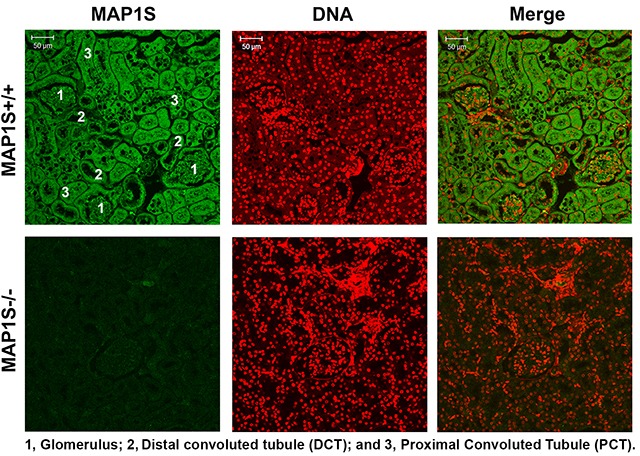
Distribution of MAP1S in mouse renal tissues Renal tissues collected from 12 month old wild-type and MAP1S^−/−^ mice were stained with anti-MAP1S antibody (green) and counter-stained with TOPO3 to visualize nuclei (red). The inserted numbers label the renal structures as indicated. Scale Bar: 50 μm.

We previously reported that MAP1S levels are low in normal mouse liver, dramatically increased upon exposure to the liver-specific carcinogen diethylnitrosamine, and then reduced to levels similar to those before exposure. After tumor foci are formed, high levels of metabolic stresses lead to an elevation of MAP1S [22]. Nothing is known about the MAP1S expression in renal tissues. In order to understand the role of MAP1S in the renal system, we first examined the expression levels of MAP1S in mouse renal tissues. Clearly, the immunostaining signal of MAP1S was strong in the glomerulus, the distal convoluted tubules and the proximal convoluted tubules of renal tissues from wild-type mice. The MAP1S signal in the MAP1S^−/−^ kidney was at the basal levels of non-specific staining (Figure 1). Levels of MAP1S expressed in renal tissues were easily detected by an antibody with high specificity.

### Levels of MAP1S in human renal cell lines and tissues from ccRCC patients

**Figure 2:**
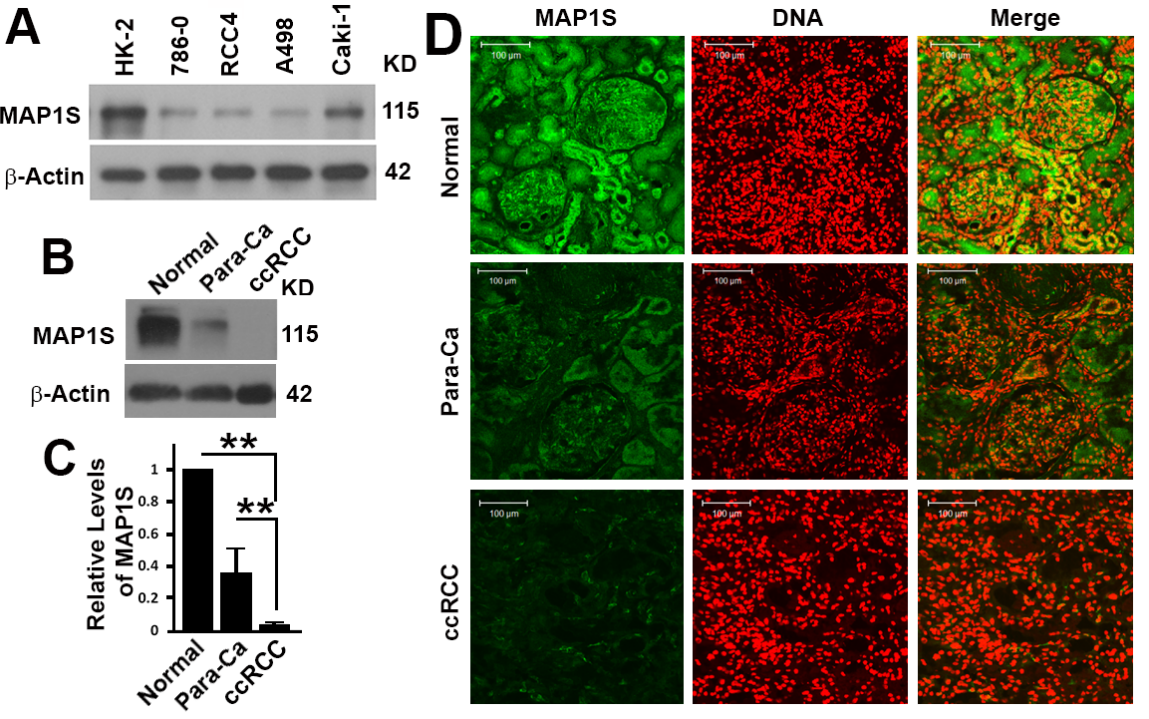
The levels of MAP1S protein in human renal cancer cell lines and cancer tissues **A.** Representative immunoblot showing the levels of MAP1S in different renal cell lines. **B.** Representative immunoblot showing the levels of MAP1S in normal, clear cell renal cell carcinoma (cc-RCC) and its adjacent tissues (para-Ca) from the same patient. **C.** The quantification of the relative intensities of MAP1S to β-Actin as shown in (B) in tissue samples collected from 8 patients. **D.** Representative images showing the immuno-florescent staining of MAP1S and nuclear DNA in the normal, para-Ca and cc-RCC tissues from the same patients.

We utilized the MAP1S antibody to test the levels of MAP1S in cultured cells. HK-2 (human kidney 2) is an immortalized proximal tubular cell line derived from a normal kidney. Levels of MAP1S in HK-2 cells were the highest, while levels of MAP1S were dramatically reduced in all other cell lines developed from human primary ccRCC (786-0, RCC4, A498 and Caki-1) (Figure 2A). Examining the levels of MAP1S in freshly collected renal tissue samples by immunoblot revealed that levels of MAP1S were the highest in normal tissues and the lowest in ccRCC (Figure 2B,2C). The same trends were confirmed by immune-florescent staining: the intensities of MAP1S in normal tissues were the highest, tissues adjacent to tumors the second, and tumor tissues the lowest (Figure 2D).

**Figure 3:**
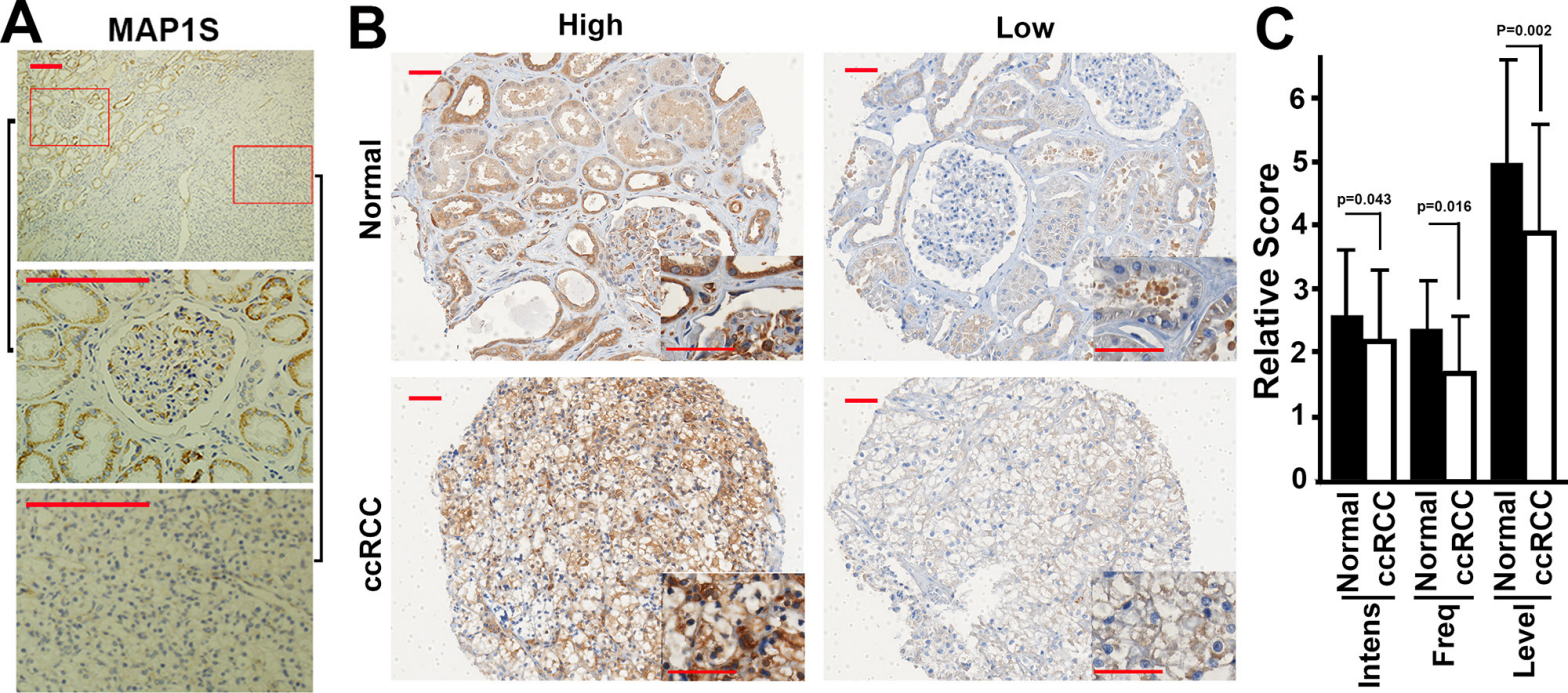
The levels of MAP1S in fixed tissues from ccRCC patients **A.** Representative images showing the immunostaining patterns of MAP1S in tissues from the same section of a ccRCC patient. The bottom two panels were the enlarged images of the red boxed areas shown in the top panel. Scale bar, 200 μm. **B.** Representative images showing low or high levels of MAP1S in ccRCC and adjacent normal tissues. Scale bar, 50 μm. **C.** A quantitation of the levels of MAP1S in ccRCC patients. Bars represent the relative score of MAP1S intensity (Intens), frequency (Freq) and level between normal and ccRCC tissues.

We further examined the levels of MAP1S in fixed tissues collected from 72 patients with different clinical and pathological characteristics (Table 1). Immunostaining the fixed tissue sections from human ccRCC patients revealed that levels of MAP1S were lower in the ccRCC than in the adjacent normal tissues on the same section (Figure 3A). Levels of MAP1S in ccRCC and normal tissues exhibited variation among different patients (Figure 3B). In general, the ccRCC tissues exhibited significant lower intensities of MAP1S staining, lower frequencies of MAP1S-positive cells and lower levels of MAP1S than their adjacent normal tissues (Figure 3C, Table 2). The examination of ccRCC tissues with high levels of MAP1S in detail revealed that the staining of MAP1S in clear cells were much weaker than other cells in the tumor focus (Figure 3B, Insert). Therefore, levels of MAP1S in ccRCC were dramatically reduced.

**Table 1:** Clinical and pathological characteristics of ccRCC patients

Classification	No.
Sex	
Female	42
Male	30
Tumor Stage	
pT1	32
pT2	24
pT3	9
pT4	7
Lymph Node Metastasis	
Negative	58
Positive	14
Distant Metastasis	
Negative	63
Positive	9
Stage	
I	30
II	18
III	12
IV	12
Tumor Grade	
G1	25
G2	31
G3	16
Average Age (years)	62.9 ± 11.0
Average Survival Time (months)	66.1 ± 30.6

**Table 2:** Clinical features of patients with ccRCC and the levels of MAP1S in their tumors

Characteristics	No.^1^	MAP1S Levels^2^	P Value^3^
**Tissue Type**			
Normal	34	5.00 ± 1.60	0.002
ccRCC	72	3.86 ± 1.73	
**Age (years)**			
≤65	35	3.54 ± 1.95	0.118
>65	37	4.14 ± 1.67	
**Sex**			
Female	42	4.17 ± 1.68	0.093
Male	30	3.43 ± 1.96	
**Tumor Stage**			
pT1, pT2	56	3.79 ± 1.82	0.523
pT3, pT4	16	4.13 ± 1.89	
**Tumor Grade**			
G1, G2	56	4.13 ± 1.64	0.021
G3	16	2.94 ± 2.17	
**Lymph Node Metastasis**			
No	58	4.17 ± 1.79	0.003
Yes	14	2.57 ± 1.40	
**Distant Metastasis**			
No	63	4.17 ± 1.62	0.000
Yes	9	1.57 ± 1.73	
**Stage Grouping**			
I, II	48	4.56 ± 1.54	0.000
III, IV	24	2.46 ± 1.53	
**Survival Time (Months)**			
>65	44	4.50 ± 1.51	0.000
≤65	28	2.86 ± 1.84	

1 Numbers of patients in each group.

2 The mean ± standard deviation of MAP1S levels in tumor tissues between different groups estimated by histological analysis.

3 The significance is estimated by Student's T Test with twotailed distribution and unequal variance.

### Higher levels of MAP1S predict a better cumulative survival

**Figure 4:**
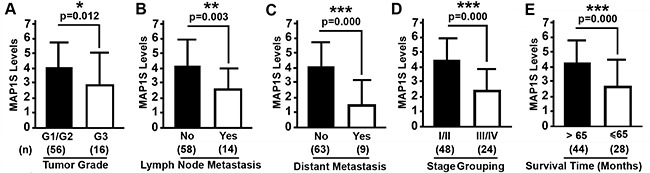
Relation between MAP1S levels and clinical features of ccRCC patients **A–E.** Comparative plots of MAP1S levels of ccRCC patients between different tumor grades (A), different states of lymph node metastasis (B), different states of distant metastasis (C), different stage grouping (D), and different survival times (E). Bars represent mean ± standard deviation of MAP1S levels between different groups. Significance is estimated by Student's T Test with two-tailed distribution and unequal variances. (n) showing the number of patients. *, p < 0.05; **, p < 0.01; ***, p<0.001.

**Figure 5:**
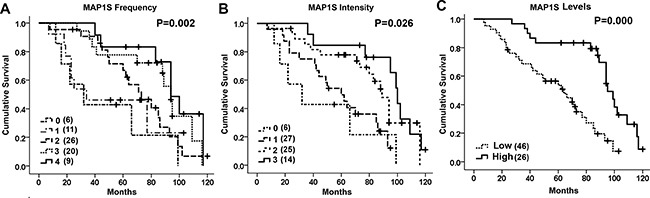
The Kaplan-Meier survival curves **A–C.** The curves showing the cumulative survival time of ccRCC patients with different frequencies (A), different intensities (B), and high or low levels of MAP1S (C). In panel A, 0, no cell; 1, 1–10%; 2, 11–50%; 3, 51–80%; and 4, >80% cells were stained. In panel B, 0, no staining; 1, cells were weakly; 2, moderately; and 3, strongly stained. In panel C, high, ≥4; and low <4 in their combined scores for intensity and frequency. Case number for each group is provided inside the parentheses. The significance of difference between two groups was estimated by chi-square test.

There was no significant difference in the levels of MAP1S between patients of different age groups, between males and females and among different tumor stages (Table 2). However, patients with G3 tumors (Figure 4A), with lymph node metastasis (Figure 4B), with distant metastasis (Figure 4C), with stage III and IV disease (Figure 4D) and with survival shorter than 65 months (Figure 4E) had significantly lower levels of MAP1S than those with G1 and G2 tumor grades, without lymph node metastasis and distant metastasis, with stage I and II, and with a survival longer than 65 months (Table 2). It has been reported that high levels of MAP1S predict better prognosis for patients suffering from prostate adenocarcinomas [23]. We further examined the relation between the levels of MAP1S and cumulative survival times of ccRCC patients. Patients with higher intensities of MAP1S staining, higher frequencies of MAP1S-positive cells and higher levels of MAP1S had significantly longer survival times (Figure 5). Thus, high levels of MAP1S predicted a better prognosis.

### A deficiency of MAP1S stimulates an accumulation of lipid droplets in the TCMK-1 mouse normal renal epithelial cells

**Figure 6:**
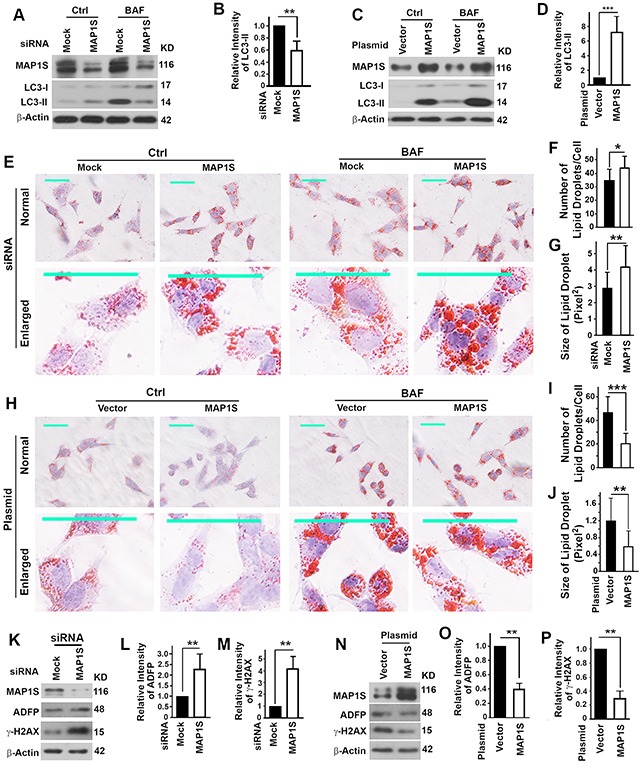
Impact of MAP1S on the autophagy clearance of lipid-droplets and genome instability in the oleate-treated mouse normal renal epithelial cell TCMK-1 **A–D.** The impact of the suppression of MAP1S with siRNA (A, B) or the overexpression of MAP1S by transfection with a plasmid **(C,D)** on the autophagy flux represented by the levels of LC3-II in the absence (Ctrl) or presence of bafilomycin A1 (BAF). Representative immunoblots (A,C) and the quantification of LC3-II levels in the presence of bafilomycin A1 (B,D) were shown. **E-J.** The impact of suppression (E-G) or overexpression (H-J) of MAP1S on the accumulation of lipid-droplets detected by Oil Red O staining. Representative images in the absence or presence of bafilomycin A1 (E,H) and the quantification of the number of lipid droplets per cell (F,I) and the size of lipid droplet in the absence of bafilomycin A1 (G,J) are shown. In E and H, top panels, normal view; bottom panels, enlarged view. Scale bar, 50 μm. **K–P.** The impact of suppression (K-M) or overexpression of MAP1S (N-P) on levels of ADFP and γ-H2AX. Representative immunoblots (K,N) and the quantification of ADFP (L,O) or γ-H2AX (M,P) were shown. Data are mean and standard deviation of at least three repeats and differences are tested with Student's T-test. **, P≤0.01.

To understand the relationship between levels of MAP1S and malignancies and survivals of ccRCC patients, we tested the impact of MAP1S on the levels of lipid droplets in the oleate-treated TCMK-1 cells. Knocking down the expression of MAP1S led to an impairment of autophagy flux as indicated by the reduced LC3-II levels in the presence of lysosomal inhibitor bafilomycin A1 (Figure 6A,6B). On the contrary, an overexpression of MAP1S caused an activation of autophagic flux (Figure 6C,6D). We observed that more lipid-droplets accumulated when cells were treated with bafilomycin A1; more and larger lipid-droplets accumulated in the cells with MAP1S suppressed (Fig. 6E-G) but more and larger in the cells overexpressing MAP1S in the absence of bafilomycin A1 (Figure 6H–6J). We observed the same trends using ADFP as an additional marker to quantify the amount of lipid droplets (Figure 6K,6L,6N,6O). In addition, a suppression of MAP1S enhanced (Figure 6K,6M) but an overexpression of MAP1S reduced the levels of γ-H_2_AX representing the DNA damage and genomic instability (Figure 6N,6P). Therefore, MAP1S activated the autophagy turnover of lipid droplets and suppressed the genomic instability.

## DISCUSSION

Whether autophagy enhances or suppresses tumorigenesis remains controversial [26, 27]. Defective autophagy stimulates accumulation of dysfunctional mitochondria and induces oxidative stress leading to apoptotic cell death [28–33]. Under such circumstances, an activated autophagy may prevent cells from death and promote the survival of cancer cells. However, autophagy suppresses oxidative stress and pyroptosis, another form of cell death. If autophagy suppresses pyroptosis, the activation of autophagy in cancer cells may reduce the pro-inflammatory stress from cancer cells to host structural, hematopoietic and immune-competent cells which surround and contain the cancer cells. Thus, the initiation, development and metastasis of cancers will be suppressed by the host cells and the patient survival will be prolonged.

Our previous results showed that MAP1S is an activator of autophagic flux. A deficiency of MAP1S causes defects in autophagy and enhances the initiation and development of hepatocellular carcinomas [19, 22]. High levels of MAP1S are associated with low malignancies of prostate cancer and predict a better survival of prostate cancer patients [23]. Here, our results similarly show that high levels of MAP1S are associated with low degrees of malignancy and distant metastasis of ccRCC, and predict a better survival of ccRCC patients.

Lipid droplets are subcellular organelles coated with ADFP for storage of triacylglycerol converted from excess free fatty acids and glucoses in plasma, and degraded through the autophagy-lysosome system [2]. LC3-II is involved in the entire process from the formation to the degradation of cytosolic lipid droplets [34]. Both ATG5 and ATG7 promote the conversion of LC3-I to LC3-II so their deletion leads to a reduction in the levels of LC3-II that is required for the formation of cytosolic lipid droplets. Although the autophagy degradation of lipid droplets is also impaired, no significant amount of lipid droplets accumulates [35, 36]. MAP1S interacts with both LC3-I and LC3-II and promotes autophagic flux [19]. Either overexpression or suppression of MAP1S does increase the levels of LC3-II, which might lead to an enhanced accumulation of lipid droplets if MAP1S only acted to increase the levels of LC3-II. However, the overexpression and suppression of MAP1S have opposite impacts on the accumulation of lipid droplets in the absence of Bafilomycin A1, suggesting that MAP1S acts mainly as a promoter of autophagic flux to enhance the degradation of lipid droplets. The depletion of MAP1S leads to accumulation of lipid droplets that enhance generation of inflammatory mediators and reactive oxygen species and induce profibrotic responses [37]. Oxidative stress and profibrotic responses promote DNA double strand breakage as indicted by γ-H_2_AX. DNA double strand breaks induce chromosomal breakage and genome instability. Cycles of cell divisions accompanying the fibrosis-induced tissue repairs facilitate the amplification of genome instability and eventually lead to tumorigenesis [38, 39]. Cancer cells may spread from their primary site to distant parts of the body through the bloodstream or the lymph system where the host immune systems work to stop the spreading [40, 41]. Inflammatory responses triggered by autophagy defects cause the death of immune cells through pyroptosis, and eventually lead to the breakdown of host immuno-response system and metastasis of cancer cells [14]. Therefore, MAP1S enhances the autophagic flux to suppress tumorigenesis and metastasis, and prolong survival of patients possibly by means of a suppression of pyroptosis.

## MATERIALS AND METHODS

### Collection of murine renal tissue

Animal protocols were approved by the Institutional Animal Care and Use Committee, Institute of Biosciences and Technology, Texas A&M Health Science Center. All animals received humane care according to the criteria outlined in the “Guide for the Care and Use of Laboratory Animals” prepared by the National Academy of Sciences and published by the National Institutes of Health (NIH publication 86-23 revised 1985). Wild-type (MAP1S^+/+^) and MAP1S knockout (MAP1S^−/−^) mice were generated and amplified in a C57BL/6 background as described in detail in our previous publication [19]. Twelve month-old male mouse littermates were sacrificed to collect renal tissues for immunofluorescent analysis with a confocal microscopy as described [42].

### Culture of renal cells for immunoblot analyses

HK-2 (ATCC^®^ CRL-2190™) is a human papillomavirus 16 (HPV-16) transformed proximal tubular cell line derived from a normal kidney. Cell line 786-0 (ATCC^®^ CRL-1932™), RCC4, A-498 (ATCC^®^ HTB-44™) and Caki-1 (ATCC^®^ HTB-46™) are derived from a human primary ccRCC. Cells were cultured using standard techniques and harvested for immunoblot analyses as previously described [42].

### Induction of lipogenesis with oleate

TCMK-1 (ATCC® CCL-139™) cells derived from mouse normal renal epithelial cells were cultured similarly to other human cell lines as previously described [42]. Cells were transfected with random or MAP1S-specific siRNA, or with control plasmid or plasmid expressing MAP1S for 24 hrs, treated with 0.25 mM oleate for 24 hours to induce lipogenesis in the absence or presence of 10 nM bafilomycin A1. Cells were then stained with 0.3% Oil Red O for 30 minutes to visualize lipid droplets and further counterstained with alum haematoxylin to visualize nuclei. The number of lipid droplets per cell and the average size of each lipid droplet were quantified as described [43].

### Enrollment of patients and collection of human renal tissue samples

This study was approved by the institutional review boards of all participating sites, and these sites provided the necessary institutional data and shared agreements before study initiation. Totally, 72 patients with ccRCC were selected from patients enrolled in the Fifth Affiliated Hospital of Guangzhou Medical University, Nanfang Hospital of Southern Medical University, Guangzhou General Hospital of Guangzhou Military Command and The University of Texas MD Anderson Cancer Center. The patients underwent treatments during the period from 2004 to 2006 and were followed for 8 years and a complete set of clinical data including age, gender, tumor stage, lymph node metastasis, distant metastasis, tumor grade, stage grouping and survival times were collected. Tumors were confirmed histopathologically and staged according to TNM classification by UICC [44, 45]. All pairs of tumors and their adjacent normal tissues were taken from patients and fixed in 10% formalin, embedded in paraffin, sectioned consecutively at 5 μm, and stained by hematoxylin and eosin. The histological types were assigned by two independent clinical pathologists in a double-blinded manner. Additionally, fresh tissues including the ccRCC tumors, renal tissues close to the tumor foci (para-Ca) and normal tissues distant from the tumor foci were collected from eight ccRCC patients enrolled during 2014. A portion of samples were frozen and used to prepare cell lysates for immunoblot analyses. Other portions were fixed and subjected to immuno-fluorescent analyses. Immunoblot analyses, immunohistochemistry analyses and immuno-fluorescent confocal microscopy were conducted following similar protocols we previously described [22, 23, 46].

### Semi-quantitative analysis

Immunostaining results were presented as percentage of positively stained tumor cells and staining intensity similarly as described [23, 46, 47]. Briefly, the percentage of positive cells was scored as 0 if no cells were stained, 1 if 1–10% cells stained, 2 if 11–50% cells stained, 3 if 51–80% cells stained and 4 if >80% cells stained. The staining intensity was scored as 0 if no staining was seen, 1 if cells were weakly stained, 2 if they were moderately stained and 3 if they were strongly stained. The combined scores for intensity and frequency were added together (0 to 7) and classified as high if the score ≥ 4 or low level if the score <4. Both percentage of positive cells and staining intensity were evaluated in a double-blinded manner.

### Statistical analysis

The statistical analyses of the differences of staining scores of MAP1S between or among various clinicopathological groups were evaluated with the Student's T-test. The cumulative survival was measured from the time of surgery until the end of the observation period and analyzed by the Kaplan-Meier method. The SAS software was used for all statistical analyses and a *P* value of <0.05 was considered significant. All statistical analyses were carried out as previously described [23, 47].

## References

[1] Mizushima N, Levine B, Cuervo AM, Klionsky DJ (2008). Autophagy fights disease through cellular self-digestion. Nature.

[2] Singh R, Kaushik S, Wang Y, Xiang Y, Novak I, Komatsu M, Tanaka K, Cuervo AM, Czaja MJ (2009). Autophagy regulates lipid metabolism. Nature.

[3] Mizushima N, Noda T, Yoshimori T, Tanaka Y, Ishii T, George MD, Klionsky DJ, Ohsumi M, Ohsumi Y (1998). A protein conjugation system essential for autophagy. Nature.

[4] Liu L, McKeehan WL, Wang F, Xie R (2012). MAP1S enhances autophagy to suppress tumorigenesis. Autophagy.

[5] Hornung V, Bauernfeind F, Halle A, Samstad EO, Kono H, Rock KL, Fitzgerald KA, Latz E (2008). Silica crystals and aluminum salts activate the NALP3 inflammasome through phagosomal destabilization. Nat Immunol.

[6] Liu L, Trimarchi JR, Smith PJ, Keefe DL (2002). Mitochondrial dysfunction leads to telomere attrition and genomic instability. Aging Cell.

[7] Mishra PK, Raghuram GV, Panwar H, Jain D, Pandey H, Maudar KK (2009). Mitochondrial oxidative stress elicits chromosomal instability after exposure to isocyanates in human kidney epithelial cells. Free Radic Res.

[8] D'Angiolella V, Santarpia C, Grieco D (2007). Oxidative stress overrides the spindle checkpoint. Cell Cycle.

[9] Li M, Fang X, Baker DJ, Guo L, Gao X, Wei Z, Han S, van Deursen JM, Zhang P (2010). The ATM-p53 pathway suppresses aneuploidy-induced tumorigenesis. Proc Nat Acad Sci USA.

[10] McClintock B (1942). The Fusion of Broken Ends of Chromosomes Following Nuclear Fusion. Proc Nat Acad Sci USA.

[11] Lamkanfi M, Dixit VM (2014). Mechanisms and functions of inflammasomes. Cell.

[12] Ryter SW, Mizumura K, Choi AM (2014). The Impact of Autophagy on Cell Death Modalities. Internat J Cell Biol.

[13] Yu J, Nagasu H, Murakami T, Hoang H, Broderick L, Hoffman HM, Horng T (2014). Inflammasome activation leads to Caspase-1-dependent mitochondrial damage and block of mitophagy. Proc Nat Acad Sci USA.

[14] Terlizzi M, Casolaro V, Pinto A, Sorrentino R (2014). Inflammasome: cancer's friend or foe?. Pharma Therapeut.

[15] Mann SS, Hammarback JA (1994). Molecular characterization of light chain 3. A microtubule binding subunit of MAP1A and MAP1B. J Biol Chem.

[16] Mann SS, Hammarback JA (1996). Gene localization and developmental expression of light chain 3: a common subunit of microtubule-associated protein 1A(MAP1A) and MAP1B. J Neurosci Res.

[17] Schoenfeld TA, McKerracher L, Obar R, Vallee RB (1989). MAP 1A and MAP 1B are structurally related microtubule associated proteins with distinct developmental patterns in the CNS. J Neurosci.

[18] Kabeya Y, Mizushima N, Ueno T, Yamamoto A, Kirisako T, Noda T, Kominami E, Ohsumi Y, Yoshimori T (2000). LC3, a mammalian homologue of yeast Apg8p, is localized in autophagosome membranes after processing. EMBO J.

[19] Xie R, Nguyen S, McKeehan K, Wang F, McKeehan WL, Liu L (2011). Microtubule-associated protein 1S (MAP1S) bridges autophagic components with microtubules and mitochondria to affect autophagosomal biogenesis and degradation. J Biol Chem.

[20] Li W, Zou J, Yue F, Song K, Chen Q, McKeehan WL, Wang F, Xu G, Huang H, Yi J, Liu L (2015). Defects in MAP1S-mediated autophagy cause reduction of mouse lifespans especially when fibronectin is overexpressed. Aging Cell.

[21] Vandin F, Clay P, Upfal E, Raphael BJ (2012). Discovery of Mutated Subnetworks Associated with Clinical Data in Cancer. Pac Symp Biocomput.

[22] Xie R, Wang F, McKeehan WL, Liu L (2011). Autophagy enhanced by microtubule- and mitochondrion-associated MAP1S suppresses genome instability and hepatocarcinogenesis. Can Res.

[23] Jiang X, Zhong W, Huang H, He H, Jiang F, Chen Y, Yue F, Zou J, Li X, He Y, You P, Yang W, Lai Y, Wang F, Liu L (2015). Autophagy Defects Suggested by Low Levels of Autophagy Activator MAP1S and High Levels of Autophagy Inhibitor LRPPRC Predict Poor Prognosis of Prostate Cancer Patients. Mol Carcinog.

[24] Frew IJ, Moch H (2015). A clearer view of the molecular complexity of clear cell renal cell carcinoma. Annu Rev Pathol.

[25] Liu K, Czaja MJ (2013). Regulation of lipid stores and metabolism by lipophagy. Cell Death Different.

[26] Kondo Y, Kanzawa T, Sawaya R, Kondo S (2005). The role of autophagy in cancer development and response to therapy. Nat Rev Can.

[27] Klionsky DJ, Abdalla FC, Abeliovich H, Abraham RT, Acevedo-Arozena A, Adeli K, Agholme L, Agnello M, Agostinis P, Aguirre-Ghiso JA, Ahn HJ, Ait-Mohamed O, Ait-Si-Ali S, Akematsu T, Akira S, Al-Younes HM (2012). Guidelines for the use and interpretation of assays for monitoring autophagy. Autophagy.

[28] Cryns V, Yuan J (1998). Proteases to die for. Gene Develop.

[29] Desagher S, Martinou JC (2000). Mitochondria as the central control point of apoptosis. Trend Cell Biol.

[30] Suen YK, Fung KP, Choy YM, Lee CY, Chan CW, Kong SK (2000). Concanavalin A induced apoptosis in murine macrophage PU5–1. 8 cells through clustering of mitochondria and release of cytochrome c. Apoptosis.

[31] Thomas WD, Zhang XD, Franco AV, Nguyen T, Hersey P (2000). TNF-related apoptosis-inducing ligand-induced apoptosis of melanoma is associated with changes in mitochondrial membrane potential and perinuclear clustering of mitochondria. J Immunol.

[32] De Vos K, Goossens V, Boone E, Vercammen D, Vancompernolle K, Vandenabeele P, Haegeman G, Fiers W, Grooten J (1998). The 55-kDa tumor necrosis factor receptor induces clustering of mitochondria through its membrane-proximal region. J Biol Chem.

[33] Boya P, Gonzalez-Polo RA, Casares N, Perfettini JL, Dessen P, Larochette N, Metivier D, Meley D, Souquere S, Yoshimori T, Pierron G, Codogno P, Kroemer G (2005). Inhibition of macroautophagy triggers apoptosis. Mol Cell Biol.

[34] Shibata M, Yoshimura K, Tamura H, Ueno T, Nishimura T, Inoue T, Sasaki M, Koike M, Arai H, Kominami E, Uchiyama Y (2010). LC3, a microtubule-associated protein1A/B light chain3, is involved in cytoplasmic lipid droplet formation. Biochem Biophys Res Commun.

[35] Shibata M, Yoshimura K, Furuya N, Koike M, Ueno T, Komatsu M, Arai H, Tanaka K, Kominami E, Uchiyama Y (2009). The MAP1-LC3 conjugation system is involved in lipid droplet formation. Biochem Biophys Res Commun.

[36] Baerga R, Zhang Y, Chen PH, Goldman S, Jin S (2009). Targeted deletion of autophagy-related 5 (atg5) impairs adipogenesis in a cellular model and in mice. Autophagy.

[37] Kiss E, Kranzlin B, Wagenblabeta K, Bonrouhi M, Thiery J, Grone E, Nordstrom V, Teupser D, Gretz N, Malle E, Grone HJ (2013). Lipid droplet accumulation is associated with an increase in hyperglycemia-induced renal damage: prevention by liver X receptors. Amer J Pathol.

[38] Kundu JK, Surh YJ (2012). Emerging avenues linking inflammation and cancer. Free Rad Biol Med.

[39] Moss SF, Blaser MJ (2005). Mechanisms of disease: Inflammation and the origins of cancer. Nat Clin Pract Oncol.

[40] Chiang AC, Massague J (2008). Molecular basis of metastasis. New England J Med.

[41] Kitamura T, Qian BZ, Pollard JW (2015). Immune cell promotion of metastasis. Nat Rev Immunol.

[42] Zou J, Yue F, Jiang X, Li W, Yi J, Liu L (2013). Mitochondrion-associated protein LRPPRC suppresses the initiation of basal levels of autophagy via enhancing Bcl-2 stability. Biochem J.

[43] Singh R, Kaushik S, Wang Y, Xiang Y, Novak I, Komatsu M, Tanaka K, Cuervo AM, Czaja MJ (2009). Autophagy regulates lipid metabolism. Nature.

[44] Greene FL, Sobin LH (2009). A worldwide approach to the TNM staging system: collaborative efforts of the AJCC and UICC. J Surg Oncol.

[45] Storkel S, Eble JN, Adlakha K, Amin M, Blute ML, Bostwick DG, Darson M, Delahunt B, Iczkowski K (1997). Classification of renal cell carcinoma: Workgroup No. 1. Union Internationale Contre le Cancer (UICC) and the American Joint Committee on Cancer (AJCC). Cancer.

[46] Ding Y, Chen B, Wang S, Zhao L, Chen J, Ding Y, Chen L, Luo R (2009). Overexpression of Tiam1 in hepatocellular carcinomas predicts poor prognosis of HCC patients. Internat J Cancer.

[47] Jiang X, Li X, Huang H, Jiang F, Lin Z, He H, Chen Y, Yue F, Zou J, He Y, You P, Wang W, Yang W, Zhao H, Lai Y, Wang F (2014). Elevated levels of mitochondrion-associated autophagy inhibitor LRPPRC are associated with poor prognosis in patients with prostate cancer. Cancer.

